# An acute phase protein ready to go therapeutic for sepsis

**DOI:** 10.1002/emmm.201303524

**Published:** 2014-01-09

**Authors:** Sofie Vandevyver, Lien Dejager, Roosmarijn E Vandenbroucke, Claude Libert

**Affiliations:** 1 Inflammation Research Center, VIBGhent, Belgium; 2 Department for Biomedical Molecular Biology, Ghent UniversityGhent, Belgium

## Abstract

The so-called Acute Phase Response (APR) begins between 12 and 48 h after the initiation of inflammation. This conserved response mainly involves the production of a set of Acute Phase Proteins (APPs) and the downregulation of other proteins (negative APPs) by hepatocytes. Although the precise identity of the APPs may differ between species, some APPs are common in all mammals.

The APR is regulated by two major players, glucocorticoids and Interleukin-6 (IL-6). Indeed, most APP coding genes contain glucocorticoid receptor responsive (GRE) elements as well as nuclear factor for IL-6 expression (NF-IL6) responsive elements (Cray *et al*, 2009). Also, other family members of IL-6, for example IL-11, can induce the APR. APPs are curious in several ways. First, their production is remarkably slow. Second, they are very diverse, and except for a few, their biological activities are poorly understood. And third, the function of the APR remains unclear. Conserved APPs include C-reactive protein (CRP), alpha-1-acid glycoprotein, alpha-1-antitrypsin, serum amyloid A, serum amyloid P, and alpha-2-macroglobulin (A2MG). Some of these proteins are hardly detectable in healthy mammals but are induced 10–1000-fold during inflammation. Because many of these APPs are heavily glycosylated (by liver enzymes that also behave like APPs), they are very stable. That is why some APPs are ideal biomarkers of inflammation, of which CRP is a typical and well-known example (Pepys & Hirschfield, 2003).

The slow production of APPs and the known biological activities of some of them indicate that they form a negative feedback loop involved in containing pathogens and limiting the inflammatory cascade. For example, alpha-1-acid glycoprotein has strong anti-inflammatory activities only at high concentrations (thus with poor specificity), and its mechanism of action is unknown but could be based on inhibition of white blood cell over-activation (Libert *et al*, 1994). Other APPs have more defined biological activities but they have to be given in high concentrations to have anti-inflammatory effects. Alpha-1-antitrypsin, the major neutrophil elastase inhibitor is one of them (Libert *et al*, 1996).

**…** some APPs are ideal biomarkers of inflammation

Sepsis is a form of systemic inflammatory response syndrome (SIRS) caused by an overwhelming reaction to infection, usually by bacteria. Recent studies estimate that 19 million people are affected by sepsis yearly, but the true number may be much higher (Angus & van der Poll, 2013). Since sepsis causes about 30% mortality, novel therapeutic interventions are urgently needed. The recent withdrawal of activated protein C (Xigris, Eli Lilly) from the market and the discontinuation of a new clinical trial with an anti-TNF antibody illustrate the difficulties for finding new effective therapeutics. Basically, sepsis displays typical inflammatory features along with activation of clotting, but also fibrinolysis and immune suppression, and many mechanisms have been suggested. Nevertheless, it is clear that an optimal host response requires that neutrophils interact with blood endothelial cells and travel to the infection site to contain the pathogens.

In this issue, Dalli *et al* (2013) report that a well-known APP, A2MG (Rehman *et al*, 2013), is an essential player in the host response to sepsis and has therapeutic potential. A2MG is a huge glycoprotein of about 750 kDa with 4 identical subunits and anti-protease activities towards all major classes of proteases, including bacterial ones. A2MG also binds many cytokines, such as TNF, IL-6, IL-1β and a receptor called low-density lipoprotein receptor-related protein-1 (LRP-1), which leads to production of very important molecules such as platelet activating factor and other bioactive lipids. Anti-inflammatory activities of A2MG have been known for a long time, for example from work with A2MG-knockout mice, and even in models of systemic inflammation (Hochepied *et al*, 2002).

The study of Dalli *et al*, however, takes the knowledge on A2MG a step further. The authors found that the plasma of sepsis patients contains lipid microparticles enriched in A2MG, presumably produced by neutrophils (Fig 1). Interestingly, surviving sepsis patients have more such A2MG enriched microparticles than non-surviving patients, suggesting that they are involved in protective pathways, and that they have a diagnostic value. Administration of such particles (either purified or prepared in the lab) to mice led to significant protection in the golden model of mouse sepsis research, the cecal ligation and puncture model (CLP), a surgical model leading to a septic, lethal peritonitis (Dejager *et al*, 2011). The authors describe reduced inflammation, better anti-bacterial responses, less hypothermia, and less mortality in the A2MG microparticles-treated mice compared to control. They report that *in vivo* knockdown of the A2MG receptor LRP-1 reduces the protective effects of A2MG. Although one can argue that this knockdown study might not be specific or strong or ubiquitous enough and that it should be corroborated by knockout techniques, the data strongly suggest that the effects of A2MG are transmitted by this receptor. In fact, the authors found evidence that microparticles-derived A2MG is captured on the cell membrane of endothelial cells, where it can bind to LRP-1 on neutrophils, which enhances the adhesion, chemotaxis and antibacterial effects of these cells. It is noteworthy that in sepsis neutrophils often fail to move to the infectious site because their chemotaxis is reduced by declining expression of CXCR2, an essential chemotaxis receptor, but A2MG prevented this decline. Finally, in sepsis, a pro-inflammatory signature is often accompanied by immune-suppressive features. Many of the failed clinical interventions addressed inflammatory mediators exclusively, but reversing the immune-suppressive status, e.g. using interferon-gamma (IFNγ), is emerging as another necessary intervention. Microparticles containing A2MG not only reduced inflammation, but also increased the production of IFNγ locally and systemically.

**Figure 1:**
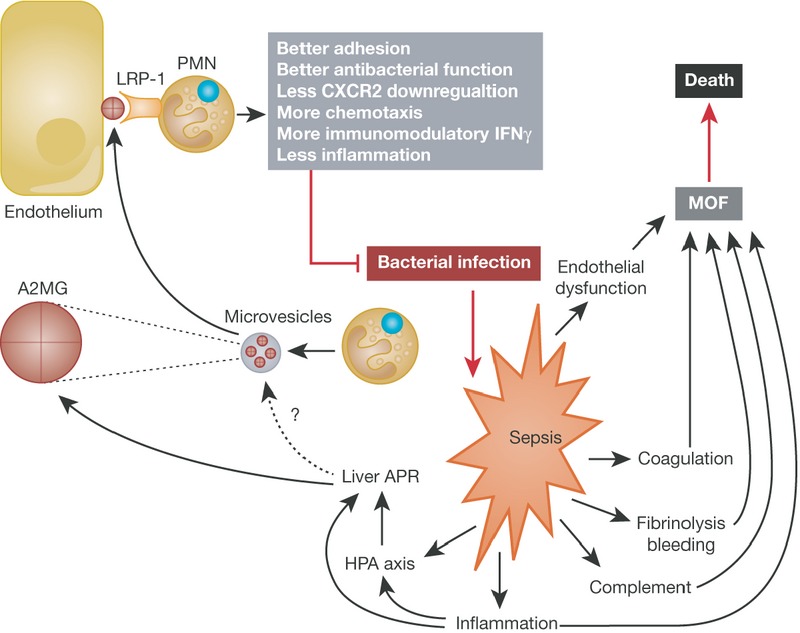
In sepsis, several complex systems are activated, such as the coagulation, fibrinolysis and complement systems, as well as inflammation and endothelial dysfunction. Multiple organ failure (MOF) is the result and death often the end point. Several feedback rescue systems are also activated. Inflammation and the activation of the hypothalamic-pituitary-adrenal axis (HPA) will activate the acute phase response (APR), leading to production of APPs such as A2MG. Dalli *et al* show that neutrophils (PMN) are activated to move to the infectious burden but also to produce microparticles, which may also contain A2MG (Dalli *et al*, 2013). The question mark means that it is uncertain whether the liver produces A2MG in microvesicles, as neutrophils do. A2MG is concentrated on the surface of endothelial cells, bind to LRP-1 on PMNs and stimulate them to help protecting against sepsis.

**…** a well-known APP, A2MG, is an essential player in the host response to sepsis and has therapeutic potential.

In summary, it is clear that microparticles-associated A2MG have powerful activities against sepsis and that the approach of the authors might prove to be an interesting new strategy for treatment of patients. The paper also confirms the recent insights that suggest that secretion of microparticles is a fast, powerful and efficient way for cell-to-cell communication in times of stress (El Andaloussi *et al*, 2013). Several questions remain unanswered however and deserve further attention. The authors suggest several protective mechanisms, such as listed in Fig 1, and one wonders which one is the most important or dominant. Also, since A2MG is a well-known APP, could the microparticles be also produced by hepatocytes as well as by neutrophils? The authors showed that microparticles associated A2MG are superior in protecting against sepsis than free, soluble A2MG, and so it would be interesting to know whether pharmacokinetics, different binding to LRP-1, or alternative mechanisms are responsible for this finding. Since A2MG is such a huge protein, a screen for other, smaller ligands of LRP-1 with similar activities may lead to even better therapeutics. Finally, one ponders whether A2MG also has therapeutic potential in other types of systemic inflammation, i.e. in severe trauma, burns, bleeding or ischemia/reperfusion.

## Conflict of interest

The authors declare that they have no conflict of interest.
